# Corrections

**DOI:** 10.1016/S1473-3099(18)30363-3

**Published:** 2018-07

*Mackenzie GA, Hill PC, Sahito SM, et al. Impact of the introduction of pneumococcal conjugate vaccination on pneumonia in The Gambia: population-based surveillance and case-control studies.* Lancet Infect Dis *2017; https://doi.org/10.1016/S1473-3099(17)30321-3—*The open access license for this Article has been corrected to CC BY 4.0. This correction been made to the online version as of June 20, 2018.

